# Natural products isolated from *Tetragonula carbonaria* cerumen modulate free radical-scavenging and 5-lipoxygenase activities in vitro

**DOI:** 10.1186/s12906-017-1748-6

**Published:** 2017-04-26

**Authors:** Karina D. Hamilton, Peter R. Brooks, Steven M. Ogbourne, Fraser D. Russell

**Affiliations:** 1 Inflammation and Healing Research Cluster, Faculty of Science, Health, Education and Engineering, School of Health and Sport Sciences, University of the Sunshine Coast, Maroochydore, QLD 4558 Australia; 2 GeneCology Research Centre, Faculty of Science, Health, Education and Engineering, University of the Sunshine Coast, Maroochydore, QLD Australia

**Keywords:** Propolis, *Tetragonula carbonaria*, Inflammation, 5-lipoxygenase, Free radical-scavenging

## Abstract

**Background:**

Propolis and cerumen are plant-derived products found in honeybees and stingless bees, respectively. Although propolis is an ancient folk medicine, the bioactivities of cerumen obtained from Australian native stingless bees (*Tetragonula carbonaria*) have not been widely studied. Therefore, we investigated selected anti-oxidant and anti-inflammatory properties of *T. carbonaria* cerumen.

**Methods:**

A methanolic extract was prepared from the combined cerumen of 40 *T. carbonaria* hives, and HPLC was used to screen for chemical constituents that scavenged 2,2-azobis(2-methylpropionamidine) dihydrochloride (AAPH). The ability of cerumen extracts to scavenge 1,1-diphenyl-2-picrylhydrazyl (DPPH) and to interfere with leukotriene B_4_ (LTB_4_) production in ionomycin-stimulated human neutrophils was also examined.

**Results:**

The extract dose-dependently scavenged DPPH (EC_50_ = 27.0 ± 2.3 μg/mL); and inhibited the 5-lipoxygenase (5-LOX)-mediated oxidation of linoleic acid (IC_50_ = 67.1 ± 9.6 μg/mL). Pre-treatment of isolated human neutrophils with the methanolic cerumen extract additionally inhibited the ionomycin-stimulated production of LTB_4_ from these cells (IC_50_ = 13.3 ± 5.3 μg/mL). Following multi-solvent extraction, the free radical-scavenging and 5-LOX-inhibiting activities of the initial cerumen extract were retained in a polar, methanol-water extract, which contained gallic acid and a range of flavonone and phenolic natural products.

**Conclusions:**

The findings identify free radical scavenging activity, and interference by extracts of *T. carbonaria* cerumen in 5-LOX–LTB_4_ signaling. Further investigation is needed to determine whether the extracts will provide therapeutic benefits for medical conditions in which oxidative stress and inflammation are implicated, including cardiovascular disease and impaired wound healing.

## Background

Propolis is a resinous, plant-derived natural product of honeybees; made by foraging for plant resins and combining these with beeswax and salivary secretions [1]. Cerumen is a similar material produced by stingless bees of the Meliponini tribe [2]. Cerumen and propolis contain chemical constituents that protect the hive against bacterial infection and opportunistic pests [1, 2]. Although propolis and cerumen typically comprise 50% plant resin, 30% beeswax, 10% essential and aromatic oils, 5% pollen and 5% organic debris [1, 3], their exact chemical compositions may vary widely.

Propolis treatment has traditionally been indicated for a wide range of ailments, which has more recently been attributed to the broad anti-oxidant, anti-cancer, anti-bacterial, anti-viral, anti-inflammatory and wound-healing effects of its extracts (reviewed in [1, 4]). These properties have often been correlated with a relatively small number of compounds in the sample or extract, including caffeic acid phenethyl ester (CAPE), artepillin C, kaempferol and galangin [5–8]. However, other studies have identified novel constituents within propolis extracts that are responsible for some of its observed bioactivities [9–12].

*Tetragonula carbonaria* is a stingless bee species native to Australia and commonly inhabits the Eastern coastline of southern Queensland and northern New South Wales [13]. *T. carbonaria* produce cerumen using the resins of the turpentine tree (*Syncarpia glomulifera*) [14] and Cadaghi gum (*Corymbia torelliana*) [15, 16]. Our research group has shown that a methanolic extract of *T. carbonaria* cerumen comprises polar constituents including gallic acid, amyrins, *C*-methyl flavanones and phloroglucinols, amongst others [17–19]. This extract, which has a chemical profile distinct to typical honeybee propolis [17], elicited a vasorelaxant response in pre-contracted human and porcine artery preparations [20] and exerted antibacterial activity against *Staphylococcus aureus* [18, 19]. An ethanolic extract of *T. carbonaria* cerumen inhibited 5-lipoxygenase (5-LOX) activity in a cell-free assay [17], although the kinetics for this response was not determined. Following on from this work, the aim of the present study was to investigate additional anti-oxidant and anti-inflammatory properties of *T. carbonaria* cerumen extracts. In particular, cell-free assays tested the potential of cerumen extracts to scavenge free radicals and inhibit the pro-inflammatory enzyme, 5-lipoxygenase (5-LOX). Stimulated human neutrophils additionally served as an in vitro model of human inflammation, to test the effects of a *T. carbonaria* cerumen extract on the 5-LOX mediated-production of the pro-inflammatory eicosanoid, leukotriene B_4_ (LTB_4_).

## Methods

### Cerumen collection and methanolic extraction

Cerumen collected from 40 *T. carbonaria* hives in the Brisbane region of South-East Queensland, Australia, was washed with water to remove debris and homogenised into one bulk sample. Raw *T. carbonaria* cerumen was extracted in 10 mL methanol and 5 mL hexane (per gram) with tumbling at 15 rpm and 22 °C for 24 h. Following paper filtration, waxes contained in the upper hexane extract were discarded, and the remaining methanolic extract was evaporated under nitrogen gas (N_2_) and freeze-dried overnight. Dried extract was reconstituted in dimethyl sulfoxide (DMSO; 1-500 μg/mL) for activity testing.

### Multi-solvent extraction of the methanolic cerumen extract

**Fig. 1:**
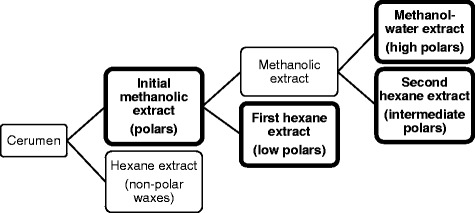
Multi-solvent extraction of *T. carbonaria* cerumen. Cerumen was partitioned into extracts of increasing polarity. Extracts in *bold *were collected, dried and tested for free radical-scavenging activity and 5-LOX inhibition

Hexane (15 mL) was added to the initial methanolic extract (30 mL), and the ‘first’ hexane extract was collected. Distilled water (15 mL) was then added to the remaining methanolic extract, which was extracted once more with hexane (20 mL). The ‘second’ hexane extract was separated from the methanol-water extract; both of which were collected. The two hexane extracts and the methanol-water extract obtained were evaporated under N_2_, freeze-dried and reconstituted in DMSO (1-5000 μg/mL) for activity testing (Fig. 1).

### Collection and isolation of human neutrophils

Neutrophils were isolated from whole blood samples collected from healthy consenting adults, who were recruited to the study through internal advertisements at the University of the Sunshine Coast (USC). Persons who had recently taken medications known to affect neutrophil function (e.g. NSAIDs and, glucocorticoids), ingested alcohol or had undertaken strenuous exercise 48 h prior to blood collection, smoked regularly, or were pregnant, were excluded from the study. Whole blood was collected from healthy volunteers who provided their informed consent, and with ethics approval from the USC Human Research Ethics Committee (S/12/389). Blood collection followed the guidelines of the Declaration of Helsinki and Tokyo for humans.

Venous blood (12 mL) was collected from the median cubital vein of four consenting individuals (24-62 years) into K_2_EDTA tubes. Neutrophils were obtained by layering 5 mL of whole blood onto 5 mL of Polymorphprep solution (Axis-Shield; Oslo, Norway) and centrifuging at 500×*g* for 30 min. The clear blood fraction containing neutrophils was collected into another centrifuge tube containing 6 mL of ‘20% media’ (Media 199 containing 20% foetal bovine serum (FBS), 50 μg/mL penicillin/streptomycin and 2 mM Glutamax-I) and centrifuged a second time at 500×*g* for 6 min. The cell pellets were resuspended in 1.3 mL of Dulbecco’s Phosphate-Buffered Saline (PBS), with 10 μL of sample smeared onto a microscope slide and stained using Diff Quik differential dye to confirm successful isolation of neutrophils using brightfield microscopy.

### High-performance liquid chromatography (HPLC) screening of free radical-scavenging constituents

Anti-oxidant compounds within the methanolic extract of *T. carbonaria* cerumen were identified using a modified HPLC screening method [21], using 2,2-azobis(2-methylpropionamidine) dihydrochloride (AAPH) as a free radical initiator. Dried methanolic *T. carbonaria* cerumen extracts (4 mg/mL) and AAPH (160 mg/mL) were reconstituted in 1:1 MilliQ water:acetonitrile, and equal volumes of each solution were incubated at 40 °C. After 8 h, reversed-phase HPLC analysis of samples was performed using a PerkinElmer Series 200 HPLC pump and auto-sampler, with a Phenomenex Synergi 4 μm Fusion-RP 80 Å analytical column, 75 × 4.6 mm with 4 μm particles (Phenomenex, Inc.; Lane Cove, NSW, Australia). Mobile phase A (MPA) was 95:5 MilliQ water:acetonitrile and mobile phase B (MPB) was 10:90 MilliQ water:acetonitrile. Following 1 min equilibration at 100% MPA; 1.2 mL/min), samples were eluted with the following method: 100% MPA for 2 min, graded to 50:50 MPA:MPB over 10 min, 100% MPB for 20 min, 100% MPB for 10 min, graded back to 100% MPA over 5 min, 100 MPA for 3 min (total run time = 50 min). Photodiode array detection occurred at 205, 260, 290 and 340 nm. Constituents of the extract that scavenged AAPH-derived free radicals were detected by the reduction or disappearance of the peak intensity for the compound following HPLC analysis. An AAPH-negative control containing 2 mg/mL extract in 1:1 MilliQ water:acetonitrile was also included in the assay, and additional analyses confirmed that deterioration of the samples did not occur over 8 h (not shown).

### Colorimetric 1,1-diphenyl-2-picrylhydrazyl (DPPH) assay

Reconstituted extracts of *T. carbonaria* cerumen were incubated with 100 μM DPPH (prepared in methanol) for 30 min at 22 °C, then absorbance was measured at 518 nm. DPPH-scavenging activity of each sample was calculated by measuring the decline in absorbance after 30 min, and expressed as a percentage of a negative control.

### Cell-free 5-LOX assay

The inhibitory effect of *T. carbonaria* cerumen on the 5-LOX-mediated oxidation of linoleic acid was examined using a modified colorimetric assay [22]. Briefly, 10 μL of each reconstituted cerumen extract was added to 0.5 mL of Solution A (containing 10 mM 3-(dimethylamino)benzoic acid, 0.05 M disodium phosphate (Na_2_HPO_4_; pH 6.0), 500 μM linoleic acid) and 5-LOX enzyme (3.4 μg in 10 μL water), then incubated for 5 min at 22 °C. Solution B (0.5 mL), containing 10 mM 3-methyl-2-benzothiazolinone and 0.1 mg/mL haemoglobin was added and incubated for a further 5 min at 22 °C. Samples were centrifuged at 20,000×g. for 3 min at 22 °C, and the absorbance of the supernatant was measured at 590 nm. Inhibition of 5-LOX by each sample was calculated by measuring the decline in absorbance after 30 min, and expressed as a percentage of a negative control.

The effect of the methanolic cerumen extract on 5-LOX enzyme kinetics was also determined. Reconstituted extract (10 μL; final concentration 100 μg/mL) was added to equal volumes of Solution A (containing 10-500 μM linoleic acid) and Solution B, then aliquotted into a 96-well microplate. Absorbance at 590 nm was measured immediately after the addition of 5-LOX (1.7 μg in 5 μL water), and periodically over one hour. The mean maximal reaction velocity (V_max_) and Michaelis constant (K_m_) were calculated for 5-LOX activity in the absence and presence of cerumen extract.

### LTB_4_ production in isolated human neutrophils

Resuspended neutrophils (35 μL) were made up to 70 μL with Dulbecco’s PBS containing methanolic cerumen extract (final concentration 1-500 μg/mL). Cell suspensions were incubated at 37 °C in a 5% CO_2_ incubator for 20 min, then treated with 2 μM ionomycin for a further 5 min (final reaction volume 80 μL) to stimulate LTB_4_ production [23]. Samples were centrifuged at 40×*g* for 6 min at 22 °C, and LTB_4_ concentration was determined spectrophotometrically at 405 nm using 50 μL aliquots of the supernatant in a LTB_4_ ELISA, according to manufacturer’s instructions (Cayman Chemical Company; Ann Arbor, USA). Background absorbance was measured in the absence of ionomycin and subtracted from all readings. Solvent (1% DMSO) and untreated controls were included in each assay, and were without effect (not shown).

### HPLC analysis of methanol-water extract

A sample of the methanol-water extract was analysed with reversed-phase HPLC using a Synergi 4 μm Fusion-RP 80 Å, 75 × 4.6 mm column (Phenomenex Inc.; Lane Cove, NSW, Australia). MPA was 95:5 MilliQ water:acetonitrile (Honeywell Burdick and Jackson®, SA, Australia) and MPB was 10:90 MilliQ water:acetonitrile. Following 1 min equilibration (100% MPA; 1.2 mL/min), samples were separated with the following method: 100% MPA for 2 min, graded to 50:50 MPA:MPB over 10 min, graded to 100% MPB over 20 min, 100% MPB for 10 min, graded to 100% MPA over 5 min, 100% MPA for 3 min (total run time = 50 min). Detection occurred at 205, 260, 290 and 340 nm. Major constituents were identified where possible, by comparison to known compounds.

### Data analysis

Data are expressed as mean ± SEM. Data were compared using one way ANOVA with Tukey’s post-hoc test, where differences were considered significant at *P* < 0.05.

## Results

### Free radical-scavenging activities of *T. carbonaria* cerumen extracts

**Fig. 2:**
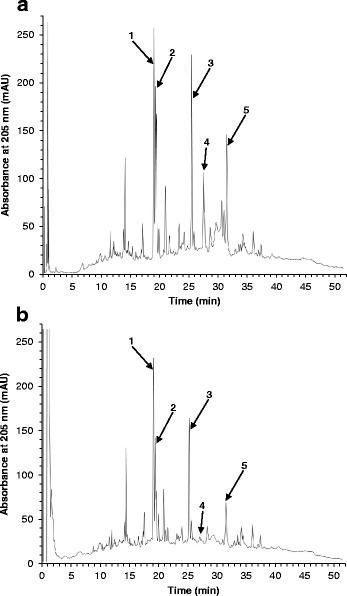
Analytical reversed-phase HPLC traces of a methanolic extract of *T. carbonaria* cerumen (2 mg/mL), in the absence **a** and presence **b** of the free radical initiator, AAPH (80 mg/mL). Peak intensities that decreased in the presence of AAPH (numbered arrows) indicate constituent compounds that scavenged AAPH-derived free radicals after 8 h

**Fig. 3:**
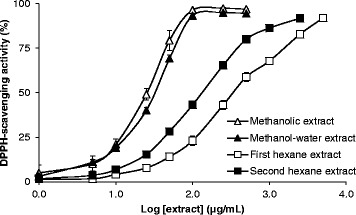
Free radical-scavenging effects of *T. carbonaria* cerumen extracts, measured using a colorimetric assay (mean ± SEM). Following multi-solvent extraction of the methanolic extract, the methanol-water cerumen extract scavenged DPPH with greater potency than the first and second hexane extracts (*P* < 0.05; *n* = 3)

Polar extracts of *T. carbonaria* cerumen displayed free radical-scavenging properties in vitro. Reversed-phased HPLC screening of the methanolic cerumen extract identified several free radical-scavenging constituents, evidenced by reduced peak intensities after reacting with the free radical initiator, APPH (Fig. 2). The extract also scavenged DPPH, in a dose-dependent manner (EC_50_ = 27.0 ± 2.3 μg/mL; Fig. 3). Following multi-solvent partitioning of the methanolic extract, DPPH-scavenging activity was identified in the resultant methanol-water and hexane extracts. However, the potency of this activity was significantly greater in the methanol-water extract (EC_50_ = 31.1 ± 1.6 μg/mL) than the first and second hexane extracts (EC_50_ = 352.2 ± 7.1 μg/mL and 128.1 ± 16.9 μg/mL respectively; *P* < 0.05).

### Effect of *T. carbonaria* cerumen extracts on 5-LOX activity and neutrophil-derived LTB_4_

**Fig. 4:**
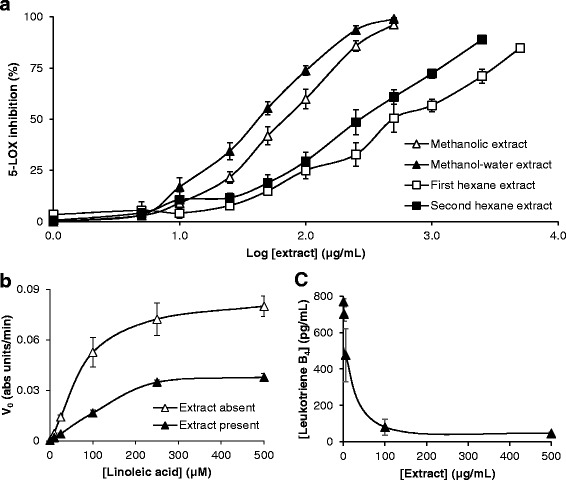
Inhibitory effects of *T. carbonaria* cerumen on the pro-inflammatory 5-LOX-LTB_4_ signaling pathway in vitro (mean ± SEM). A methanolic cerumen extract dose-dependently inhibited the 5-LOX-mediated oxidation of linoleic acid (**a**), through reduced V_max_ and increased K_m_ (**b**; *P* < 0.05; *n* = 3). The extract additionally inhibited LTB_4_ production in human neutrophils stimulated with 2 μM ionomycin (**c**; *n* = 4). Following multi-solvent extraction, the methanol-water cerumen extract inhibited 5-LOX activity with greater potency than the first and second hexane extracts (a; *P* < 0.05; *n* = 3)

Similar to its DPPH-scavenging activity, the inhibitory effect of the methanolic cerumen extract on cell-free 5-LOX activity was dose-dependent (IC_50_ = 67.1 ± 9.6 μg/mL; Fig. 4a). The kinetics of linoleic acid oxidation by 5-LOX (V_max_ = 0.08 ± 0.006 absorbance units/min; K_m_ = 71.3 ± 10.4 μM) were significantly altered in the presence of 100 μg/mL extract (V_max_ = 0.04 ± 0.002 absorbance units/min; K_m_ = 115.0 ± 7.3 μM; *P* < 0.05; Fig. 4b). The extract also inhibited ionomycin-induced LTB_4_ production in isolated human neutrophils, but with greater potency than it inhibited the 5-LOX-mediated oxidation of linoleic acid in the colorimetric assay (IC_50_ = 13.3 ± 5.3 μg/mL; Fig. 4c). Following multi-solvent extraction of the methanolic extract, the resultant methanol-water extract inhibited cell-free 5-LOX activity with comparable potency (IC_50_ = 42.8 ± 4.6 μg/mL); which was greater than the first and second hexane extracts (IC_50_ = 427.5 ± 76.2 μg/mL and 239.3 ± 40.7 μg/mL respectively; *P* < 0.05; Fig. 4a). Kojic acid, used as a standard 5-LOX inhibitor, inhibited this enzyme with high potency (pIC_50_ = 1.08 ± 0.038 μg/mL).

### Composition of a methanol-water extract of *T. carbonaria* cerumen

**Fig. 5:**
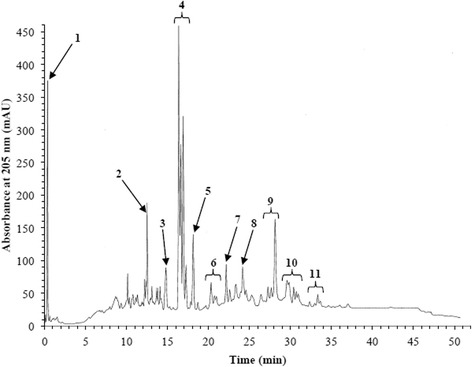
Analytical reversed-phase HPLC trace of a methanol-water extract of *T. carbonaria* cerumen. Major compounds in the areas highlighted (1-11) were identified where possible, by comparison to known compounds

Analysis of the methanol-water extract using reversed-phase HPLC revealed that it was a complex mixture of numerous compounds (Fig. 5).

By comparison to known compounds, Peak 1 was identified as gallic acid, while the remaining constituents could only be assigned to compound classes (Table 1). The majority of main compounds belong to the flavanone and phenolic classes of natural product.

**Table 1:** Major constituents in the methanol-water extract of *T. carbonaria* cerumen as determined by HPLC-MS and spectral comparison to known compounds

Major constituent	Molecular Weight	Major Fragment Ion	Identity
1	170		Gallic Acid
2	302	229	O-methyl-aromadendrin
3	422	329	Flavone
4	270	167	dihydroxydihydroflavone
5	424	285	Flavone
6	454	329	Flavone
7	540	453	Flavone
8	540	427	Flavone
9	522	387	Flavone
10	552	271	Flavone
11	386	251	Flavone

## Discussion

Propolis and cerumen are plant-derived bee products that exhibit a broad range of chemical and biological properties, regardless of their geographical origins. Although honeybee propolis is considered an ancient folk medicine, the recent popularity of alternative medicines and neutraceuticals has seen research and commercial interest in propolis and cerumen come to the fore. This study aimed to investigate selected anti-oxidant and anti-inflammatory properties of cerumen collected from Australian native stingless bees; a natural product that has not been widely studied. We subsequently found that polar extracts of *T. carbonaria* cerumen possessed potent free radical-scavenging properties, and exhibited inhibitory effects on the 5-LOX-LTB_4_ signaling pathway in vitro. The polar methanol-water extracts were, on average, 10.6- and 4.9-fold more potent that the lower polarity first hexane and second hexane extracts, respectively, in our assays.

It is widely reported that propolis of diverse global origins exert anti-oxidant effects [1, 4, 24]. In particular, propolis extracts have previously been found to scavenge reactive oxygen species (ROS) and synthetic free radicals, inhibit lipid peroxidation, reduce ferric (Fe^3+^) and cupric (Cu^2+^) ions and elicit metal-chelating effects in vitro [5, 11, 25–31]. In this study, we demonstrated that *T. carbonaria* cerumen also possessed free radical-scavenging properties, evidenced by the ability of its polar extracts to scavenge AAPH and DPPH in cell-free assays. However, whilst the anti-oxidant properties of propolis are often correlated with the phenolic acid and flavonoid content of its extracts [5, 11, 25–29, 31], *T. carbonaria* cerumen comprises a unique chemical profile. Previous gas chromatography-mass spectrometry (GC-MS) analysis of *T. carbonaria* cerumen extracts by our group showed that its chemical profile differed from New Zealand propolis and did not contain CAPE [17], a compound regarded to be largely responsible for the anti-oxidant and anti-inflammatory properties of temperate, honeybee propolis [32, 33]. Further studies are required to investigate whether *T. carbonaria* cerumen may exert similar, and additional, anti-oxidant effects in cell-based systems. Gallic acid, one of the compounds identified in cerumen, was previously reported by our group to inhibit 5-LOX activity (pIC_50_ = 5.62 ± 0.35 μg/mL) [17]. The methanol-water cerumen extract had 7.6-fold and 39.6-fold lower potency for inhibition of 5-LOX compared to gallic acid and kojic acid, respectively. Bioactivity-guided fractionation of *T. carbonaria* polar extracts are ongoing to elucidate the remainder of its bioactive constituents.

Eicosanoids such as LTB_4_ are pro-inflammatory signaling molecules produced from the enzyme-catalyzed metabolism of arachidonic acid (AA). Upon liberation from phospholipids by phospholipase A_2_, AA is oxidized to LTB_4_ via an intermediate precursor, LTA_4_, in a pathway catalyzed by 5-LOX and LTA_4_ hydrolase [34]. In the present study, we found that a methanolic extract of *T. carbonaria* cerumen inhibited the pro-inflammatory 5-LOX-LTB_4_ signaling pathway in vitro. In cell-free assays, the extract dose-dependently inhibited the 5-LOX-mediated oxidation of linoleic acid, by reducing the maximal reaction velocity and the affinity of 5-LOX to its substrate. These findings collectively suggest that the effects of the extract on 5-LOX resembled a mixture of competitive and non-competitive enzyme inhibition [35]. Using a cell-based model of human inflammation, the cerumen extract additionally suppressed LTB_4_ production by ionomycin-stimulated neutrophils. Our results coincide with others who demonstrated that an ethanol extract of Brazilian green propolis inhibited the release of cysteinyl leukotrienes, LTC_4_, LTD_4_ and LTE_4_, in peripheral leukocytes of patients with allergic rhinitis [36]. However, since the *T. carbonaria* cerumen extract inhibited neutrophil-derived LTB_4_ production with five-fold greater potency than cell-free 5-LOX activity (IC_50_ = 13.3 ± 5.3 μg/mL versus IC_50_ = 67.1 ± 9.6 μg/mL, respectively), we hypothesize that its mechanism of action may not be specific to 5-LOX inhibition. Although it is unclear whether propolis and cerumen may inhibit LTB_4_ synthesis by exerting additional effects on phospholipase A_2_ or LTA_4_ hydrolase enzymes, it has been suggested that their anti-oxidant properties may assist in suppressing eicosanoid synthesis, by non-specifically scavenging the peroxy systems implicated in AA metabolism [37].

## Conclusion

The aim of the present study was to investigate anti-oxidant and anti-inflammatory properties of polar extracts of cerumen from Australian native stingless bees, *T. carbonaria.* The study demonstrated that cerumen exerted potent free radical-scavenging effects, was a mixed enzyme inhibitor of 5-LOX, and reduced the Ca^2+^-ionophore-induced production of LTB_4_ from human neutrophils in vitro. Polar constituents of cerumen belonged primarily to flavanone and phenolic classes of compound. Further investigation is needed to determine whether the extracts will provide therapeutic benefits for medical conditions in which oxidative stress and inflammation are implicated.

## Abbreviations

5-LOX: 5-lipoxygenase
AAPH: 2,2-azobis(2-methylpropionamidine) dihydrochloride
CAPE: caffeic acid phenethyl ester
DMSO: dimethyl sulfoxide
DPPH: 1,1-diphenyl-2-picrylhydrazyl
HPLC: High-Performance Liquid Chromatography
LTB_4_: leukotriene B_4_
PBS: Dulbecco’s Phosphate-Buffered Saline
*T. carbonaria*: *Tetragonula carbonaria.*
